# Effects of dairy consumption on SIRT1 and mitochondrial biogenesis in adipocytes and muscle cells

**DOI:** 10.1186/1743-7075-8-91

**Published:** 2011-12-20

**Authors:** Antje Bruckbauer, Michael B Zemel

**Affiliations:** 1 Department of Nutrition, University of Tennessee, Knoxville, TN, USA

**Keywords:** SIRT1, Dairy, Leucine, β-Hydroxy-β-Methylbutyrate (HMB), α-ketoisocaproate (KIC), Adipose tissue, Muscle, Insulin sensitivity, Mitochondrial biogenesis

## Abstract

**Background:**

Recent data from this laboratory suggest that components of dairy foods may serve as activators of SIRT1 (Silent Information Regulator Transcript 1), and thereby participate in regulation of glucose and lipid metabolism. In this study, an *ex-vivo/in-vitro *approach was used to examine the integrated effects of dairy diets on SIRT1 activation in two key target tissues (adipose and muscle tissue).

**Methods:**

Serum from overweight and obese subjects fed low or high dairy diets for 28 days was added to culture medium (similar to conditioned media) to treat cultured adipocytes and muscle cells for 48 hours.

**Results:**

Treatment with high dairy group conditioned media resulted in 40% increased SIRT1 gene expression in both tissues (p < 0.01) and 13% increased enzyme activity in adipose tissue compared to baseline. This was associated with increased gene expression of peroxisome proliferator-activated receptor-gamma coactivator 1 alpha (PGC-1α), nuclear respiratory factor 1 (NRF1), cytochrome oxidase c subunit 7 (Cox 7), NADH dehydrogenase and uncoupling protein 2 (UCP2) in adipocytes as well as uncoupling protein 3 (UCP3), NRF1 and Cox 7 in muscle cells (p < 0.05). Further, direct incubation of physiological concentrations of leucine and its metabolites α-Ketoisocaproic acid (KIC) and β-hydroxy-methylbuteric acid (HMB) with recombinant human SIRT1 enzyme resulted in 30 to 50% increase of SIRT1 activity (p < 0.05).

**Conclusions:**

These data indicate that dairy consumption leads to systemic effects, which may promote mitochondrial biogenesis in key target tissues such as muscle and adipose tissue both by direct activation of SIRT1 as well as by SIRT1-independent pathways.

## Background

The beneficial effects of energy restriction on lifespan and protection against metabolic disease are mediated, in part, by SIRT1 (Silent Information Regulator Transcript 1) and SIRT3 in mammals and by the SIRT1 orthologue Sir2 in lower species [1]. Both SIRT1 and SIRT3 are NAD^+^-dependent class III protein deacetylases which sense the energy/nutrient status via the NAD^+^/NADH ratio [2]. While SIRT3 is mainly located in the mitochondria, SIRT1 functions as a transcriptional repressor via histone deacetylation in the nucleus. However, it also modifies the acetylation level of transcription factors such as p53, NF-κB and FOXO [1,3]. Activation of SIRT1 stimulates PGC1α, with consequent activation of mitochondrial biogenesis and metabolism [4] and associated improvements of insulin sensitivity and suppression of oxidative and inflammatory stress [5,6].

Dairy foods have been reported to have multiple effects on adipocyte and muscle metabolism and therefore play a significant role in modulating energy metabolism and obesity risk [7-9]. While some of these effects appear to be mediated by dietary calcium [10], recent evidence indicates that the high concentration of branched chain amino acids (BCAA) contribute to these effects. In particular, the BCAA leucine plays a distinct role as it has a pivotal function in protein synthesis signaling and as it appears to play an important role in the re-partitioning of dietary energy from adipose to skeletal muscle [11], although it is not clear whether these effects are mediated by intact leucine or by its' metabolites α-ketoisocaproate (KIC) and β-Hydroxy-β-Methylbutyrate (HMB).

Our recent data indicate that components of dairy foods may serve as activators of SIRT1. Leucine administration stimulated mitochondrial biogenesis and fat metabolism in both adipocytes and muscle cells; these effects were mediated, in part, by SIRT1, and SIRT1 knockdown markedly attenuated the effects [12]. In contrast calcitriol, which is increased in response to suboptimal calcium intake, inhibited mitochondrial biogenesis, increased energy efficiency and reactive oxygen species (ROS) generation [12,13]. In addition, data from our mouse microarray study suggest that dairy components, especially calcium and leucine, up-regulate SIRT1-dependent signaling pathways for fat oxidation, attenuate pathways of inflammatory response (including NF-κB signaling) and stimulate insulin signaling in skeletal muscle and adipose tissue [14]. Furthermore, consistent with reports of SIRT1 effects on lifespan, dairy-rich diets reduced the early mortality in mice [15].

**Figure 1:**
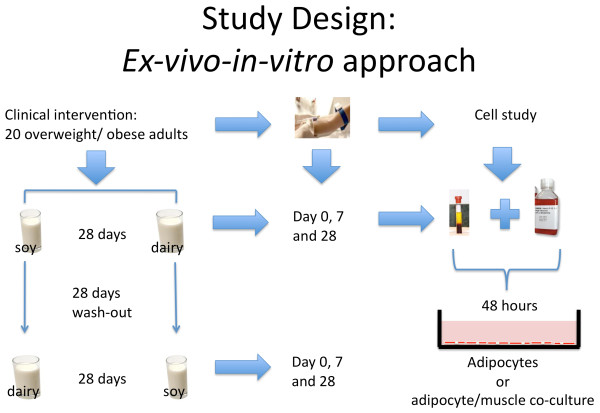
**The *ex-vivo/in-vitro *Approach**. 20 overweight or obese subjects were fed a low (soy) or high dairy diet for 28 days in a cross-over design. Blood from each intervention period was drawn at start (day 0), at day 7 and at the end (day 28) to provide serum to utilize in cell studies. The serum reflected the integrated systemic response to the diets and was then added to culture medium (as serum conditioned medium) to treat cultured human adipocytes and muscle cells, separately and in co-culture, for 48 hours in order to assess the integrated effects of dairy feeding on SIRT1 activation in two key target tissues.

These observations provided the mechanistic framework for our hypothesis that calcitriol and leucine modulation of SIRT1 in adipose tissue and skeletal muscle is the central signaling event that links the effects of calcitriol, leucine and dairy foods on fatty acid oxidation, oxidative stress, insulin sensitivity and inflammatory stress. Since adiposity is associated with changes in glucose and lipid metabolism, we tested this hypothesis in overweight and obese subjects using an *ex-vivo/in-vitro *approach in which subjects were fed low or high dairy diets to provide serum to utilize in cell studies. The serum reflected the integrated systemic response to the diets and was then added to culture medium (analogous to conditioned medium) to treat cultured human adipocytes and muscle cells, separately and in co-culture, in order to assess the integrated effects of dairy feeding on SIRT1 activation in these two key target tissues (Figure 1).

## Materials and methods

### Clinical intervention (in-vivo study)

#### Subjects

Twenty otherwise healthy adults [14 males, 6 females; 10 overweight (BMI 25.0 - 29.9) and 10 mildly obese (Stage 1 obesity; BMI 30.0-34.9)] were recruited from the faculty, staff and student populations of the University of Tennessee. Enrolled subjects were 31.0 ± 10.3 years of age. Subjects were excluded for the following reasons: BMI <25 or ≥ 30 (overweight subjects) or < 30 or ≥ 35 (obese subjects); type II diabetes requiring the use of any oral antidiabetic agent and/or insulin; history/presence of significant metabolic disease, active gastrointestinal disorders, or eating disorders; adverse response to dairy foods; recent use of tobacco, pharmacotherapeutic agents, over-the-counter anti-obesity agents, or psychotropic medication; recent initiation of an exercise program, recent initiation/change in hormonal birth control or hormone replacement therapy, or pregnancy/lactation.

#### Interventions

Two weight-maintenance diets (soy-based placebo diet (500-600 mg calcium/day) and a dairy diet (three daily servings; 1200-1400 mg calcium/day) were presented to the subject groups in a randomized crossover design. Each diet was presented for 28 days, with fasted plasma obtained at baseline (day 0), day 7 and day 28 of each dietary period. Diets were presented in random order. Half of the overweight and half of the obese subjects received the dairy-free diet first and the other half received the dairy-adequate diet first. The two dietary periods were separated by a 28-day washout period. After washout, the study was repeated with subjects exposed to the other diet for an additional 28 days. Dietary intervention in females commenced during the luteal stage (14-24 days) after the onset of menses to control for confounding of menstrual cycle and oxidative and/or inflammatory stress; this resulted in a longer >28-day washout for some subjects (up to 38 days) in order to start at the same point in the menstrual cycle for both phases of the cross-over. This study was approved from an ethical standpoint by the Institutional Review Board of the University of Tennessee-Knoxville and written informed consent was obtained from the participants of this study. This study was registered at clinicaltrials.gov (NCT00686426). The primary purpose of this study was to assess the effects of dairy versus soy on oxidative and inflammatory stress, and the results on those outcomes were recently reported [16]

#### Diets

Two diets (dairy and soy) in the form of "smoothies" were administered three times per day throughout each 28-day treatment period. Each smoothie contained 170 kcal, 10 grams protein, 1 gram fat and 30 grams carbohydrate. Two of the daily smoothies were consumed by subjects at the clinic site and the third was taken for off-site consumption. The dairy smoothies were milk-based, using non-fat dry milk as the protein source, and contained 350 mg calcium per smoothie. The placebo smoothies used soy protein isolate as the protein source and contained 50 mg calcium per smoothie. For both dairy and placebo, subjects were provided a choice of fruit flavorings. The two diets (smoothies plus other foods consumed) were constructed to provide comparable levels of macronutrient and fiber--approximating the average consumption in the U.S. (fat, ~35% of total kcal, carbohydrates ~49%, protein ~16%, fiber 8-12 g/day). Maintenance levels of calorie intake were determined from measurement of resting metabolic rate (RMR) via indirect calorimetry. Total daily energy expenditure and maintenance energy requirements were calculated as 1.2 - 1.4 X RMR, depending upon level of physical activity.

Nutritional supplements were not permitted. Caffeine intake was maintained at a constant level per subject based on baseline assessment. Subjects were given individual instruction, counseling and assessment from the study dietitian regarding dietary adherence. All subjects maintained complete diet and physical activity diaries throughout the study.

### In-vitro Study

Serum of day 0, 7 and 28 of each of the two dietary periods (dairy and soy) from six individual subjects was utilized. This provided a baseline for each dietary period and short- (7 day) and intermediate-term (28 day) assessment of the effects of diet on SIRT1. Serum was diluted with the adipocyte or myocyte culture media at 25% vol/vol and cells were incubated for 48 hours at 37°C. For each subject, gene expression analysis was performed in two cell replicates and SIRT1 activity in three cell replicates.

### Cell Culture

#### Human Adipocytes

Human omental cultured adipocytes were obtained as plated differentiated adipocytes at 2 weeks old from Zen-Bio, Inc. (Research Triangle, NC, USA), originally isolated from the omental visceral fat depot from four healthy obese female subjects (average age 40 y old, average BMI = 45.6 kg/m^2^). Cells were handled according to manufacture's instructions. Prior to each experiment, cells were transferred to a low serum medium (0.2% fetal bovine serum (FBS)) overnight. Cells were then washed with fresh serum-free medium and re-fed with serum-free adipocyte medium containing the indicated serum treatments and incubated at 37°C in 5% CO_2 _for 48 hours prior to analysis (SIRT1 activity or RNA extraction for gene expression). Cell viability was measured by trypan blue exclusion.

#### Mouse adipocytes

3T3-L1 preadipocytes were incubated at a density of 8000 cells/cm2 (10 cm2 dish) and grown in Dulbecco's modified Eagle's medium (DMEM) containing 10% FBS and antibiotics (1% penicillin/streptomycin) at 37°C in 5% CO2 in air. Confluent preadipocytes were induced to differentiate with a standard differentiation medium consisting of DMEM-F10 (1:1, v/v) medium supplemented with 10% FBS, 1 μM dexamethasone, 0.5 mM isobutylmethylxanthine, and antibiotics (1% penicillin/streptomycin). Preadipocytes were maintained in this differentiation medium for 3 days and subsequently cultured in adipocyte medium. Cultures were re-fed every 2 to 3 days to allow 90% cells to reach full differentiation. Before treatment cells were incubated in low serum medium (0.2%FBS) overnight and then washed with fresh medium, re-fed with medium containing the different serum treatments and incubated at 37°C in 5% CO2 for 48 h before analysis.

#### Mouse myocytes

C2C12 cells were incubated at a density of 8000 cells/cm2 (10 cm2 dish) and grown in Dulbecco's modified Eagle's medium (DMEM) containing 10% FBS and antibiotics (1% penicillin/streptomycin) at 37°C in 5% CO2 in air. Cells were grown to 100% confluence, changed into differentiation medium (DMEM with 2% horse serum and 1% penicillin/streptomycin), and fed with fresh differentiation medium every other day until myotubes were fully formed (6 days). Cells were then used for co-culture as described below.

#### Adipocyte-myocyte co-culture

Cells are co-cultured using transwell inserts with a 0.4 μm porous membrane (Corning) to separate adipocytes and muscle cells, as previously described [11,12,17]. Each cell type was grown independently in transwell plates and, following differentiation, inserts containing adipocytes are transferred to myotubes plates. The cells are then incubated in DMEM-medium with antibiotics containing the indicated serum treatments and incubated at 37°C in 5% CO_2 _for 48 hours, after which cells in the lower well are harvested for further analysis.

#### RNA extraction

The Ambion ToTALLY RNA isolation kit (Ambion, Inc., Austin, Tex., USA) was used to extract total RNA from cells according to the manufacturer's instruction. The concentration, purity and quality of the isolated RNA were assessed by measuring the 260/280 ratio (1.8-2.0) and 260/230 ratio (close to 2.0) by using the ND-1000 Spectrophotometer (NanoDrop Technologies Inc., Del. USA).

#### Gene Expression

Expression of adipocyte and myocyte 18S, SIRT1, PGC1-α, cytochrome c oxidase subunit VIIc1 (Cox 7c), mitochondrial NADH dehydrogenase (MT-ND1 and NDUFA), nuclear respiratory factor 1 (NRF1) and uncoupling protein (UCP2 (adipocyte)/UCP3 (myocyte) was measured via quantitative real-time PCR using an ABI 7300 Real-Time PCR system (Applied Biosystems, Branchburg, NJ) with a TaqMan^® ^core reagent kit. All primers and probe sets were obtained from Applied Biosystems TaqMan^® ^Assays-on-Demand and utilized accordingly to manufacturers instructions. Pooled RNA from each cell type was serial-diluted in the range of 0.0156 - 50 ng and used to establish a standard curve; total RNA for each unknown sample was also diluted in this range. RT-PCR reactions were performed according to the instructions of the ABI Real-Time PCR system and TaqMan Real Time PCR Core Kit. Expression of each gene of interest was then normalized using the corresponding 18S quantitation. Data for each gene is presented as a ratio to 18S.

#### SIRT1 Activity

SIRT1 activity was measured by using the SIRT1 Fluorimetric Drug Discovery Kit (BML-AK555, ENZO Life Sciences International, Inc. PA, USA). In this assay, SIRT1 activity is assessed by the degree of deacetylation of a standardized substrate containing an acetylated lysine side chain. The substrate utilized is a peptide containing amino acids 379-382 of human p53 (Arg-His-Lys-Lys[Ac]), an established target of SIRT1 activity; SIRT1 activity is directly proportional to the degree of deacetylation of Lys-382. For activator screening of SIRT1, 0.5 mM Leucine, 0.5 mM KIC or 0.05 mM HMB were added to human recombinant SIRT1 enzyme and incubated with peptide substrate (25 μM), and NAD^+ ^(500 μM) in a phosphate-buffered saline solution at 37°C on a horizontal shaker for 45 minutes. The reaction was stopped with the addition of 2 mM nicotinamide and a developing solution that binds to the deacetylated lysine to form a fluorophore. Following 10 minutes incubation at 37°C, fluorescence was read in a plate-reading fluorometer at an excitation wavelength of 360 nm and an emission wavelength of 450 nm. For endogenous SIRT1 activation, the assay was modified by using 2.5 ul of adipose or muscle cell lysate. In each assay, human recombinant SIRT1 enzyme (1 Unit per well), resveratrol (20 mM for cell lysate assays, and 10 and 2 mM for activator screening), a SIRT1 activator, and suramin sodium (5 mM), a SIRT1 inhibitor were utilized as positive and negative controls in each set of reactions. A standard curve was constructed using deactylated substrate (0-10 μM). Data for endogenous SIRT1 activation were normalized to cellular protein concentration measured via BCA-assay.

#### Statistical Analysis

Change from baseline values were computed for each outcome variable. All data were then expressed as mean percent change from baseline ± SE. Data were analyzed by one-way ANOVA, and significantly different group means (p < 0.05) were then separated by the least significant difference test using SPSS (SPSS Inc, Chicago, IL).

## Results

**Figure 2:**
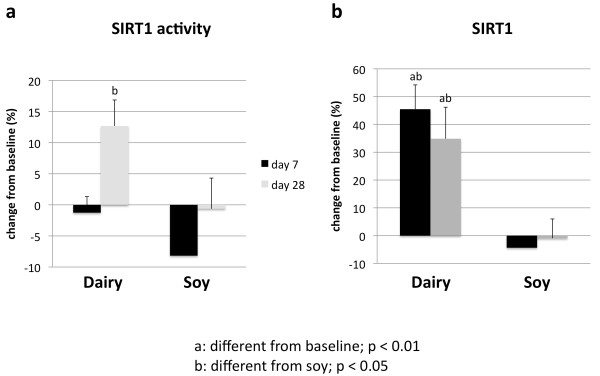
**The effects of serum treatment on SIRT1 activity (a) and SIRT1 gene expression (b) in human adipocytes**. Cultured human adipocytes were incubated for 48 hours with human serum collected on day 0 (baseline), 7 or 28 after high dairy diet or soy-based control diet. Values are expressed as means of % change to baseline ± SE (n = 5 subjects/group). Differing letters above the bars denote significant differences between groups, p ≤ 0.05.

**Figure 3:**
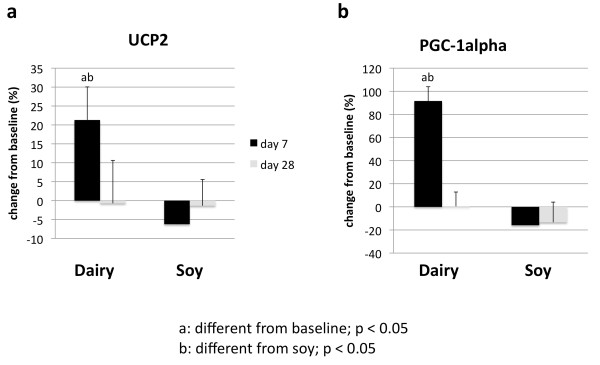
**Effects of serum treatment on mitochondrial biogenesis in human adipocytes**. Cultured human adipocytes were incubated for 48 hours with human serum collected on day 0 (baseline), 7 or 28 after high dairy diet or soy-based control diet. (a) Uncoupling protein 2 (UCP2) and (b) PGC-1α were significantly increased by dairy at day 7 compared to baseline and soy-based diet. Values are expressed as means of % change to baseline ± SE (n = 4 to 5 subjects/group). Gene expression data are normalized to 18S. Differing letters above the bars denote significant differences between groups, p ≤ 0.05.

**Figure 4:**
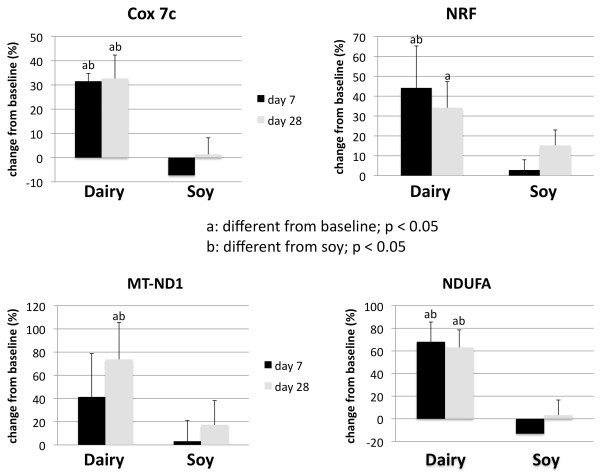
**Effects of serum treatment on mitochondrial component genes in human adipocytes**. Cultured human adipocytes were incubated for 48 hours with human serum collected on day 0 (baseline), 7 or 28 after high dairy diet or soy-based control diet. (a) Cytochrome c oxidase subunit 7 (Cox 7c), (b) nuclear respiratory factor 1 (NRF1), and mitochondrial NADH dehydrogenase ((c) mitochondrial encoded subunit (MT-ND1) and (d) nuclear encoded subunit (NDUFA)) were significantly up-regulated after dairy feeding compared to baseline and soy diet. Values are expressed as means of % change to baseline ± SE (n = 4 to 5 subjects/group). Gene expression data are normalized to 18S. Differing letters above the bars denote significant differences between groups, p ≤ 0.05.

Treatment of cultured adipocytes and myocytes with serum obtained from subjects fed a high dairy diet for 4 weeks resulted in significant changes of SIRT1 activity and gene expression as well of mitochondrial component genes. Figure 2 shows that incubation of human adipocytes with serum drawn after 28 days of high dairy treatment increased significantly both SIRT1 activity (Figure 2a) and SIRT1 gene expression (Figure 2b) by 13 and 40%, respectively, compared to baseline while the soy-based diet had no significant effect. These effects were associated with concomitant changes in mitochondrial biogenesis indicated by increased gene expression of uncoupling protein (UCP)-2 (Figure 3a) and PGC-1α (Figure 3b) by 20 and 90% (p < 0.05), respectively. In addition, mitochondrial component genes such as mitochondrial NADH dehydrogenase (Figure 4c and d), cytochrome C oxidase (Cox 7c) (Figure 4a) and nuclear respiratory factor (NRF-1) (Figure 4b) were highly up-regulated by dairy. Similar effects could be noted after treatment of mouse 3T3-L1 adipocytes with the human serum (data not shown).

**Figure 5:**
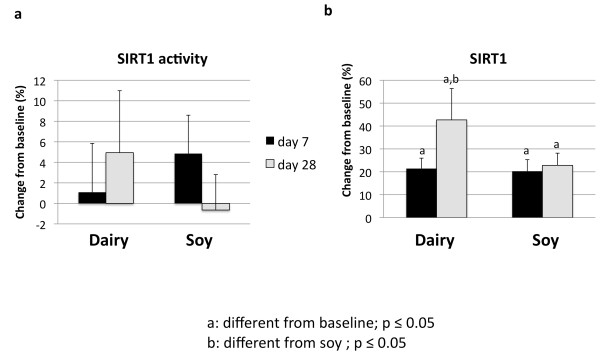
**The effects of serum treatment on SIRT1 activity (a) and SIRT1 gene expression (b) in mouse skeletal muscle**. Cultured C2C12 muscle cells were grown in co-culture with 3T3-L1 adipocytes and incubated for 48 hours with human serum collected on day 0 (baseline), 7 or 28 after high dairy diet or soy-based control diet. Values are expressed as means of % change to baseline ± SE (n = 6 subjects/group). Differing letters above the bars denote significant differences between groups, p ≤ 0.05.

**Figure 6:**
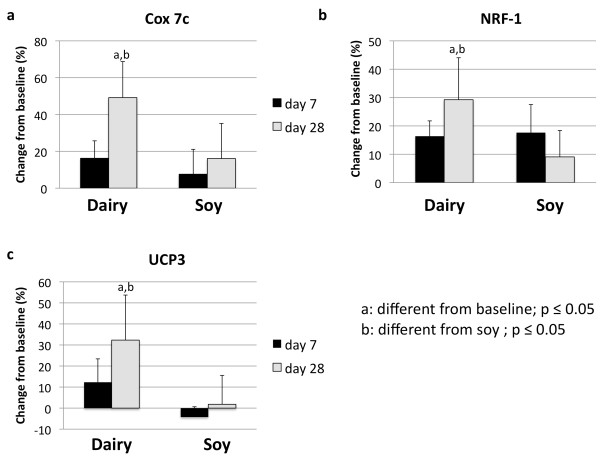
**Effects of serum treatment on mitochondrial biogenesis in mouse skeletal muscle cells**. C2C12 muscle cells were grown in co-culture with 3T3-L1 adipocytes and incubated for 48 hours with human serum collected on day 0 (baseline), 7 or 28 after high dairy diet or soy-based control diet. (a) Cytochrome c oxidase subunit 7 (COX 7), (b) nuclear respiratory factor 1 (NRF1) and (c) UCP 3 were significantly up-regulated 28 days after dairy feeding compared to baseline and soy-based control. Values are expressed as means of % change to baseline ± SE (n = 6 subjects/group). Gene expression data are normalized to 18S. Differing letters above the bars denote significant differences between groups, p ≤ 0.05.

Next, we treated C2C12 muscle cells in co-culture with 3T3-L1-adipocytes with serum obtained from the subjects after dairy or soy feeding. Dairy feeding resulted in 43% increase of SIRT1 gene expression in the muscle cells (p < 0.05), which was significantly different from baseline and soy-based control (Figure 5b). Although there was no difference detected for SIRT1 activity (Figure 5a), genes such as Cox-7c, nuclear respiratory factor (NRF)-1 and UCP3 were significantly up-regulated by 22%, 29% and 32% (p < 0.05), respectively, by treatment with serum from 28 days after dairy feeding suggesting that mitochondrial biogenesis was also stimulated in muscle cells (Figure 6, ba, and 6c).

**Figure 7:**
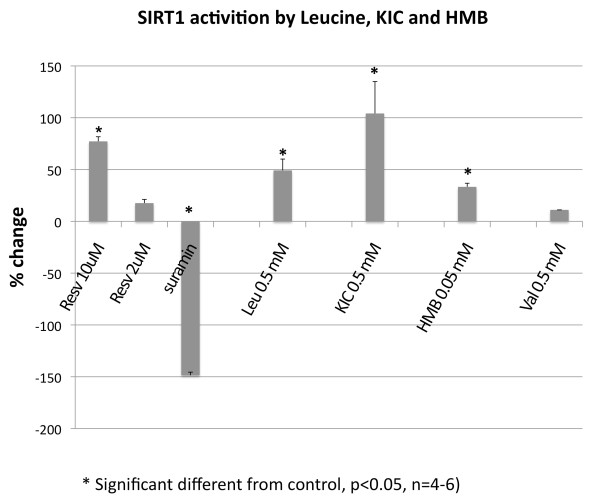
**Direct effects of Leucine, β-Hydroxy-β-Methylbutyrate (HMB) and Ketoisocaproate (KIC) on SIRT1 activity**. Recombinant human SIRT1 enzyme was incubated with Leucine, HMB and KIC for 45 min and fluorescence of deacetylated substrate was measured. Resveratrol and Suramin were used as positive and negative control, respectively. Valine was used as BCAA control. Data are expressed as means of % change of SIRT1 activity ± SE (n = 4, p ≤ 0.05).

To test, whether Leucine and its metabolites β-Hydroxy-β-Methylbutyrate (HMB) and Ketoisocaproate (KIC) are direct activators of SIRT1, we incubated these compounds with recombinant human SIRT1 enzyme and measured the activity of SIRT1 by the amount of deacetylated substrate. All three compounds were able to directly increase SIRT1 activity by 30 to 100%, which was comparable to the effects of low dose (2 to 10 μM) resveratrol while valine, used as a branched-chain amino acid control, had no effect (Figure 7).

## Discussion

This *ex-vivo/in-vitro *study indicates that high dairy feeding in humans over 4 weeks results in significant systemic changes which may induce activation of SIRT1 and downstream targets of mitochondrial biogenesis in key target tissues as demonstrated here in muscle and adipose cells. These effects may contribute to observed changes in energy metabolism and oxidative and inflammatory stress. We previously reported that the high dairy intake in this clinical study resulted in significant suppression of oxidative and inflammatory biomarkers, including reductions in plasma malondialdehyde, 8-isoprostane-F_2_, TNF-α, IL-6 and MCP-1, and increased levels of the anti-inflammatory cytokine adiponectin [16]. We have also shown that dairy components, especially calcium and leucine, up-regulated signaling pathways for fat oxidation, attenuated inflammatory pathways such as NF-κB signaling and stimulated insulin signaling in skeletal muscle and adipose tissue, suggesting a SIRT1 dependent regulation [14]. Similarly, supplemental leucine intake by addition to drinking water in mice rescued metabolic changes to a high fat diet and was associated with improvement of glucose tolerance and insulin signaling as well as decrease in adipose tissue inflammation [18]. Although the authors of this study did not assess SIRT1 activation, SIRT1 dependence is consistent with their observations. Yoshizaki et al [6] reported beneficial effects of SIRT1 activation on glucose uptake and insulin signaling, and improvement of inflammatory markers in 3T3-L1 adipocytes while SIRT1 depletion exerted the opposite effect. Moreover, modest global overexpression of SIRT1 in mice resulted in protection against metabolic damage from a high-fat diet by up-regulation of antioxidant proteins such as MnSOD and NRF1, and lower lipid-induced activation of pro-inflammatory cytokines such as TNFα and IL-6 via reduction of NF-κB [19]. These effects were manifested in lower inflammation, improved glucose tolerance and nearly complete protection from hepatic steatosis. In contrast, heterozygous SIRT1 knockout (SIRT1^+/-^) mice developed severe hepatic steatosis on high-fat diets, accompanied by lower energy expenditure and increased inflammation [20]. Accordingly, our observation of increased SIRT1 activity and expression in muscle and adipose tissue following incubation with serum from subjects fed a high dairy diet likely represents the mechanism of the observed reduction of oxidative and inflammatory stress in these subjects. However, the present data are limited by the opportunistic use of archival samples from our previous clinical trial [16]; accordingly, no direct comparison of the effects of increasing dairy food intake in lean vs. obese subjects is possible.

Mitochondrial loss and/or dysfunction play a key role in metabolic disorders such as insulin resistance, type II diabetes and cardiovascular disease [21-23], and stimulation of mitochondrial biogenesis with resveratrol increased insulin sensitivity and prevented obesity and insulin-resistance in mice fed a high-fat diet [24]. The majority of mitochondrial proteins, including most of those involved in oxidative phosphorylation, are nuclear encoded and transported into the mitochondria from the cytoplasm while only 13 protein subunits involved in electron transport are encoded in the mitochondrial genome (mtDNA) [2]. For mitochondrial biogenesis, both the nuclear and mitochondrial protein subunits have to form complexes and thus require a coordinated crosstalk and regulation for proper function. PGC-1α, a downstream-target of SIRT1, is a key regulator of mitochondrial biogenesis in response to external stimuli. Up-regulation of PGC-1α activates the expression of nuclear-encoded OxPhos components genes as well as of nuclear respiratory factor (NRF1), which also regulates the transcription of mitochondrial genes [25,26].

Although it is possible that the previously observed systemic changes in oxidative and inflammatory biomarkers [16] may contribute to increased SIRT1 expression and/or activity, we have previously demonstrated that leucine administration stimulated mitochondrial biogenesis and metabolism in skeletal muscle and adipose cells and that these effects were mediated, in part, by SIRT1 [12], while calcitriol treatment exerted the opposite effects. Similarly, we demonstrate in this study that a leucine rich diet in form of dairy results in up-regulation of PGC-1α as well as in up-regulation of downstream target genes such as NRF-1, UCP2 and 3, NADH dehydrogenase and cytochrome c oxidase indicative of stimulated mitochondrial biogenesis in muscle and adipose tissue. Although we cannot directly attribute the observed *ex vivo *effects to the leucine content of the high dairy diet, the concentrations of leucine used in our *in vitro *studies are comparable to the plasma levels typically achieved in response to leucine-rich milk or whey-based diets [27], while soy protein isolate produced only ~30% the leucine level when studied at the same protein load (10 g/dose).

Data from this study indicate that not only leucine but also its metabolites HMB and KIC are direct activators of SIRT1 enzyme. It has been suggested that some of the leucine effects may be attributed to its metabolites, which have also variable effects on protein metabolism and immune function [28]. The majority of the first step of leucine metabolism, the transamination to KIC, occurs in muscle. Orally administered alpha-KIC to food-deprived rats exerted stimulatory effects on protein-synthesis in skeletal muscle, similar to L-leucine administration, but not in liver; however, it was not clear whether these effects were direct effects of KIC or caused by the reversible conversion of KIC to leucine [29]. Earlier results demonstrated that incubation with KIC could decrease protein degradation in rat diaphragms but did not stimulate protein synthesis while leucine incubation did both [30].

Although effects of HMB supplementation on muscle strength and gain are also conflicting, HMB has been used as a therapeutical supplement for years to attenuate muscle loss and damage under various conditions [31]. Some of this anti-catabolic activity seems to be mediated by reduction of ROS formation [32]. In addition, HMB may play a role as a potentially dietary immunomodulator since it has been shown to decrease proliferation and TNF-α production in stimulated human peripheral blood monocytes by 35% [33] and suppressed NF-κB expression in tumor-bearing Wistar rats, thereby reducing tumor growth and tumor cell proliferation [34]. Since SIRT1 is a negative regulator of NF-κB [35,36] and attenuation of NF-kB activity by SIRT1 results in suppression of TNF-α [37,38], it is possible that the above mentioned effects of HMB are mediated, at least in part, by HMB activation of SIRT1. It is not yet clear how leucine or its metabolites KIC and HMB directly stimulate SIRT1 in a cell free system. We speculate that they may act as allosteric activators producing a conformational change in SIRT1, which increases binding to its substrate.

The stimulation of mitochondrial biogenesis in muscle cells was not associated with underlying changes in SIRT1 activity, although SIRT1 gene expression was up-regulated. Therefore, it is likely that SIRT1-independent pathways also modulate some of the effects of dairy components. AMP-activated protein kinase (AMPK), a key regulator of energy metabolism, is a likely target, as it also serves as an energy sensor and regulates cellular metabolism. In addition, there is a bidirectional interaction between AMPK and SIRT1; AMPK activates SIRT1 by increasing cellular NAD+ levels and, conversely, SIRT1 stimulates AMPK by activation of LKB1 [39]. Since adiponectin activates AMPK [40], and we previously demonstrated the high dairy diet to increase adiponectin (16), adiponectin stimulation of AMPK may play a significant role in the observed effects.

## Conclusions

In conclusion, we utilized an *ex vivo/in-vitro *approach to test whether high dairy feeding in people is related to underlying changes in key regulatory enzymes of metabolism and mitochondrial biogenesis in muscle and adipose tissue which may be responsible for observed alterations in inflammatory and oxidative stress markers. Our *in-vitro *results in muscle and adipose cells indicate that systemic effects of dairy feeding may result in SIRT1- activation in key target tissues such as muscle and adipose tissue. This may represent one possible central signaling event that links the effects of calcitriol, leucine and dairy foods on fatty acid oxidation, oxidative stress, insulin sensitivity and inflammatory stress. Strengths of this study include the linkage of signaling events in established cell systems to clinical dietary interventions using a novel *ex-vivo *approach and the control inherent in the randomized crossover design clinical trial. Limitations of this study include those inherent in extrapolating from *ex-vivo *to *in-vivo*, the inability of this design to discriminate among the dairy components responsible for the observed effects, and the modest sample size limiting the degree to which these findings may be generalized. In addition, we did not examine the mechanism how leucine or its metabolites directly stimulate SIRT1, and cannot exclude the possibility that binding to the fluorophore used in the SIRT1 assay contributes to the observed increase in SIRT1 activity.

## Competing interests

The authors declare that they have no competing interests.

## Authors' contributions

AB and MBZ jointly conceived of this study and participate in its design and coordination. MBZ directed the clinical study. AB conducted all cellular experiments and the cell-free SIRT 1 experiments. Both authors read and approved of the final manuscript.
